# Olfactory‐to‐Entorhinal Network Dysrhythmias Drive Parkinson's Cognitive Impairment Through Frequency‐Specific Oscillatory Decoupling

**DOI:** 10.1002/advs.202512183

**Published:** 2025-12-05

**Authors:** Shuaishuai Wang, Zhishen Cai, Xingfeng Mao, Yixuan Zhang, Yunlong Pan, Jiawen Cheng, Xuechun Wang, Hengyi Song, Sasaki Takuya, Ming Lu, Gang Hu, Xiuxiu Liu, Yingmei Lu, Feng Han

**Affiliations:** ^1^ Department of Physiology School of Basic Medical Sciences Nanjing Medical University Nanjing 211166 China; ^2^ Jiangsu Province Innovation center for Brain‐Inspired intelligence technology Nanjing 210029 China; ^3^ Medical Basic Research Innovation Center for Cardiovascular and Cerebrovascular Diseases Ministry of Education Nanjing 211166 China; ^4^ Key Laboratory of Cardiovascular & Cerebrovascular Medicine, School of Pharmacy Nanjing Medical University Nanjing 211166 China; ^5^ Department of Pharmacology Graduate School of Pharmaceutical Sciences Tohoku University Sendai 980‐8578 Japan; ^6^ Jiangsu Key Laboratory of Neurodegeneration Department of Pharmacology Nanjing Medical University Nanjing 211166 China; ^7^ Key Laboratory of Modern Toxicology of Ministry of Education Nanjing Medical University Nanjing 211166 China; ^8^ Northern Jiangsu Institute of Clinical Medicine The Affiliated Huaian No.1 People's Hospital of Nanjing Medical University Huaian 223300 China

**Keywords:** cognitive impairment, lateral entorhinal cortex, neural oscillation, olfactory bulb, Parkinson's disease

## Abstract

Parkinson's disease (PD)‐associated cognitive decline is heralded by olfactory dysfunction, but the network mechanisms bridging sensory and cognitive impairments remain poorly defined. Combining chronic multisite electrophysiology, behavioral tracking, and machine learning in PD models, a hierarchical disintegration of oscillatory dynamics across the olfactory network that mechanistically drives disease progression is uncovered. Early‐stage PD mice are identified to show attenuated odor discrimination, accompanied by hyperexcitability of mitral/tufted (M/T) cells. Causally linking these deficits, aberrant gamma oscillation in the cross‐olfactory network is identified as a causal factor underlying olfactory deficits. Notably, cognitive impairment emerged at later stages, correlating with abnormal theta oscillations in the cross‐olfactory network. Pharmacological modulation of the olfactory bulb (OB)‐lateral entorhinal cortex (LEC) pathway ameliorated cognitive deficits and restored cross‐network theta oscillation. Collectively, the findings establish cross‐olfactory network oscillations as dual diagnostic and therapeutic targets for PD cognitive impairment, providing a mechanism‐guided framework for early intervention.

## Graphical abstract

Wang *et al* reveal that in the olfactory system, the selective impairment of gamma‐driven Barcode features underlying early olfactory deficits, while the selective impairment of theta‐driven Barcode features underlying later cognitive deficits, further establishing cross‐network gamma oscillations in the early stage as a biomarker of later cognitive deficits in Parkinson's disease pathogenesis.

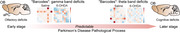

## 1 Introduction

Parkinson's disease (PD), while traditionally characterized by its motor symptoms, poses a profound clinical challenge due to its non‐motor manifestations, particularly cognitive impairment that develops in advanced stages and currently lacks effective treatment.^[^ ^1^, ^2^, ^3^ ^]^ While dopamine replacement therapy effectively manages motor symptoms, cognitive decline as a robust predictor of dementia remains untreatable, underscoring the urgent need for targetable mechanisms.^[^ ^4^, ^5^ ^]^ Current diagnostic approaches fail to detect PD‐related cognitive impairment during the pre‐symptomatic phase, and the underlying pathophysiology especially the spatiotemporal evolution of neural circuit dysfunction remains poorly understood.

The olfactory dysfunction appears years before motor symptoms in PD patients,^[^ ^6^, ^7^ ^]^ yet its causal relationship with cognitive decline remains largely unexplored, representing a significant gap in our understanding of PD progression.^[^ ^8^, ^9^ ^]^ Growing evidence suggests that olfactory deficits are mechanistically linked to subsequent cognitive decline, with pathological changes observed in olfactory‐processing regions such as the piriform and entorhinal cortex also implicated in neurodegenerative cognitive disorders.^[^ ^10^, ^11^, ^12^ ^]^ While odor identification tests and neuroimaging biomarkers show potential for PD diagnosis,^[^ ^13^ ^]^ fundamental questions remain regarding the precise temporal progression of olfactory dysfunction and the development of standardized biomarkers for disease staging, limiting their clinical utility.

Recent advances in neurophysiology have established electrophysiological signatures as powerful, functionally relevant biomarkers for neurological disorders.^[^ ^14^, ^15^ ^]^ Oscillatory patterns in local field potentials have proven particularly valuable in epilepsy and Alzheimer's disease, where they reflect disease‐specific circuit disturbances.^[^ ^16^, ^17^, ^18^, ^19^ ^]^ This success highlights the considerable, yet unrealized potential of electrophysiological approaches in PD, where oscillatory abnormalities in cognitive networks remain poorly characterized. The olfactory bulb (OB) serves as the primary processing center in the olfactory system. Its main output neurons, mitral and tufted (M/T) cells, act as a critical relay, integrating sensory information and transmitting it to higher olfactory regions such as the piriform cortex and entorhinal cortex.^[^ ^20^ ^]^ Specifically, the dynamic relationship between olfactory circuit activity and PD‐associated cognitive impairment representing a critical intersection of spatial and temporal disease progression has not been systematically investigated, leaving a major gap in our understanding of PD pathophysiology.

Here, we hypothesize that cross‐olfactory network oscillations encode modifiable targets for PD cognitive decline. By combining in vivo electrophysiology, circuit‐specific pharmacology, and machine learning, we decode the spatiotemporal hierarchy linking early olfactory gamma anomalies to delayed theta‐mediated cognitive deficits. Our approach establishes a causal framework for sensory‐cognitive network disintegration in PD, directly informing clinical biomarker and neuromodulation strategies.

## 2 Results

### 2.1 Olfactory Deficits Precede Cognitive Impairment in PD Mice

**Figure 1:**
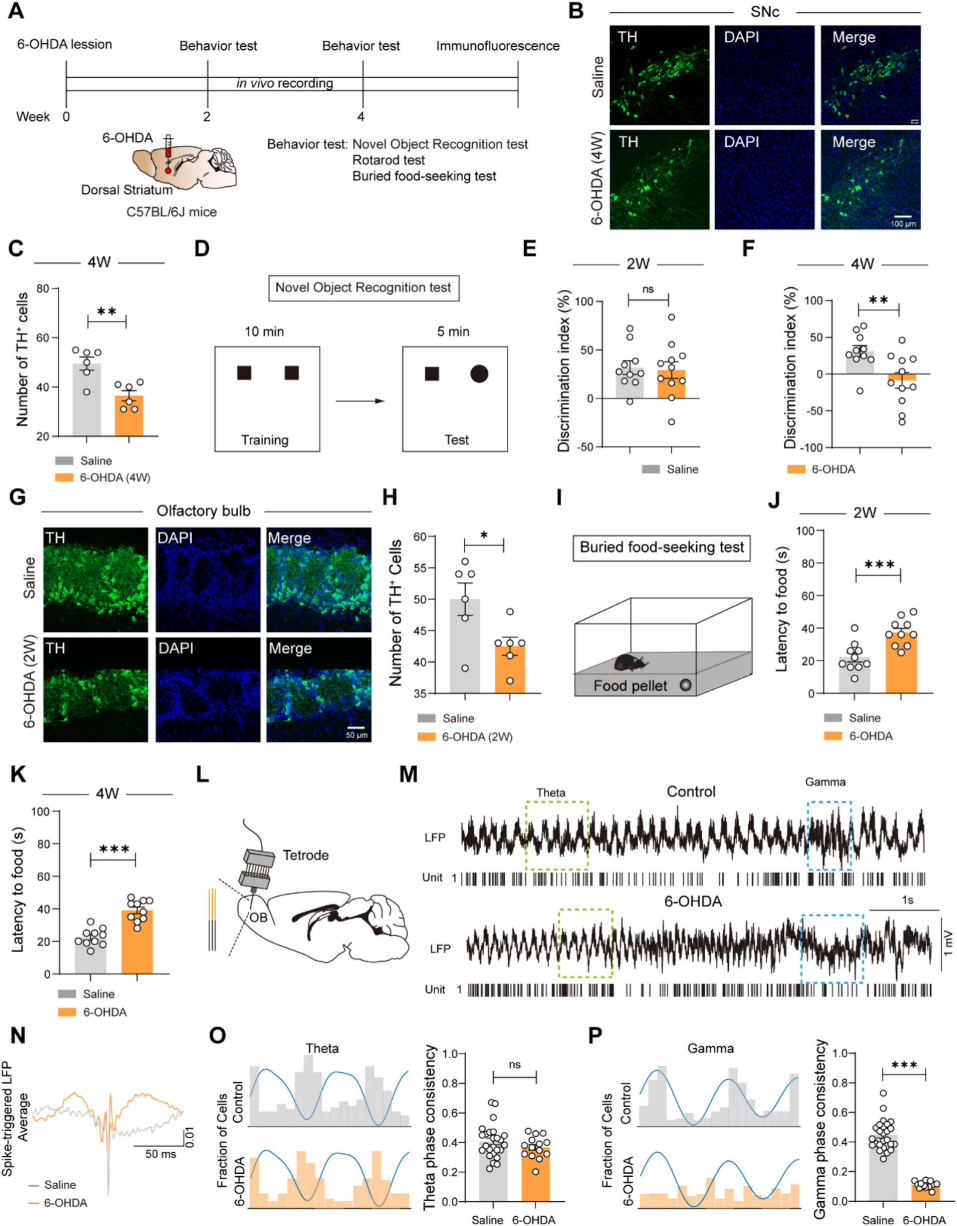
Olfactory deficits precede cognitive impairment in PD mice. A) Schematics and timeline of the experimental procedures. 6‐OHDA is injected into DSTr. B) Representative fluorescent images of TH^+^ neurons in SNc. C) Quantification of TH^+^ neurons in SNc (n = 6 in each group, and 2 views of slices were provided for each mouse). D) Behavioral procedure for the novel object recognition test. E,F) Novel object preference index at 2 weeks (E) and 4 weeks (F) post‐6‐OHDA injection, Saline: n = 10 mice, 6‐OHDA: n = 11 mice. G) Representative fluorescent images of the TH^+^ neuron in OB; H) Quantification of TH^+^ neurons in OB (n = 6 in each group, and 2 views of slices were provided for each mouse). I) Behavioral procedure for the buried food‐seeking test. J,K) The latency to find food 2 (J) and 4 (K) weeks after 6‐OHDA injection, Saline: n = 10 mice, 6‐OHDA: n = 10 mice. L) Schematics of in vivo recording procedures. M) Representative raster plot and LFP raw data. N) Schematic illustrations of spike‐triggered LFP. O,P) Analysis of spike‐triggered theta (O) and gamma (P) consistency (Saline: n = 26 units from 5 mice, 6‐OHDA: n = 15 units from 4 mice). Data are presented as mean ± SEM; ^*^ *P* < 0.05, ^**^ *P* < 0.01, ^***^ *P* < 0.001; ns, not significant. Unpaired two‐tailed Student's t test for C, E,F, H, J,K, O,P (right).

We induced Parkinsonian pathology by unilateral 6‐hydroxydopamine (6‐OHDA) injections into the dorsal striatum (dSTR) of mice. All experiments were conducted between 2–4 weeks post‐injection (**Figure** **1**A). Immunohistochemical analysis confirmed significant dopaminergic neuron loss in the substantia nigra pars compacta (SNc), with 6‐OHDA mice showing a 26.3% reduction in tyrosine hydroxylase‐positive (TH^+^) cells compared to controls at 4 weeks (*p* = 0.0033, Figure 1B,C). This neurodegeneration was accompanied by motor deficits in the rotarod test at 4 weeks (Latency to fall: saline 244.2 ± 10.27 s vs 6‐OHDA 133.5 ± 12.90 s, *p* < 0.0001), while motor function remained unaffected 2 weeks post‐injection (Figure S1A–C, Supporting Information). Cognitive impairment, assessed by novel object recognition test (NOR), emerged specifically at 4 weeks (discrimination index: saline 31.06 ± 7.90% vs 6‐OHDA −8.50 ± 10.58%, *p* = 0.0083), but not at 2 weeks (Figure 1D–F).

Strikingly, olfactory system pathology preceded these motor and cognitive deficits. At 2 weeks post‐injection, we observed a 15% reduction in TH^+^ neurons within the glomerular layer in OB (*p* = 0.0293, Figure 1G,H), accompanied by functional olfactory impairment in the buried food‐seeking test (Latency to food: saline 22.1 ± 2.78 s vs 6‐OHDA 37.3 ± 2.61 s at 2 weeks, *p* = 0.0009; 22.5 ± 1.68 s vs 39.0 ± 2.06 s at 4 weeks, *p* < 0.0001, Figure 1I,K). Performance in the visible pellet test remained intact across groups (Figure S1D–F, Supporting Information), thereby confirming that the deficit observed in the olfactory‐based buried pellet test was not attributable to impairments in vision, motor function, or motivation.

To investigate the neural basis of these olfactory deficits, we performed simultaneous single‐unit and local field potential (LFP) recordings in the OB (Figure 1L,M). Cluster analysis identified two distinct neuronal populations (Figure S2A–C, Supporting Information). 6‐OHDA mice showed significant mitral cell hyperexcitability, with a nearly 2‐fold increase in spontaneous firing rate (*p* = 0.023, Figure S2D,E, Supporting Information). Spike‐triggered LFP average analysis revealed a reduction of the spike‐triggered LFP power in 6‐OHDA mice compared with controls (Figure 1N). Despite preserved theta‐phase locking (*p* = 0.258, Figure 1O), we observed markedly reduced gamma‐phase locking (75.3% reduction, *p* < 0.0001, Figure 1P), indicating disrupted excitatory synchrony in OB networks that likely impairs sensory processing precision.

### 2.2 Gamma Oscillations Mediate Odor Discrimination Deficits in Early‐Stage PD

**Figure 2:**
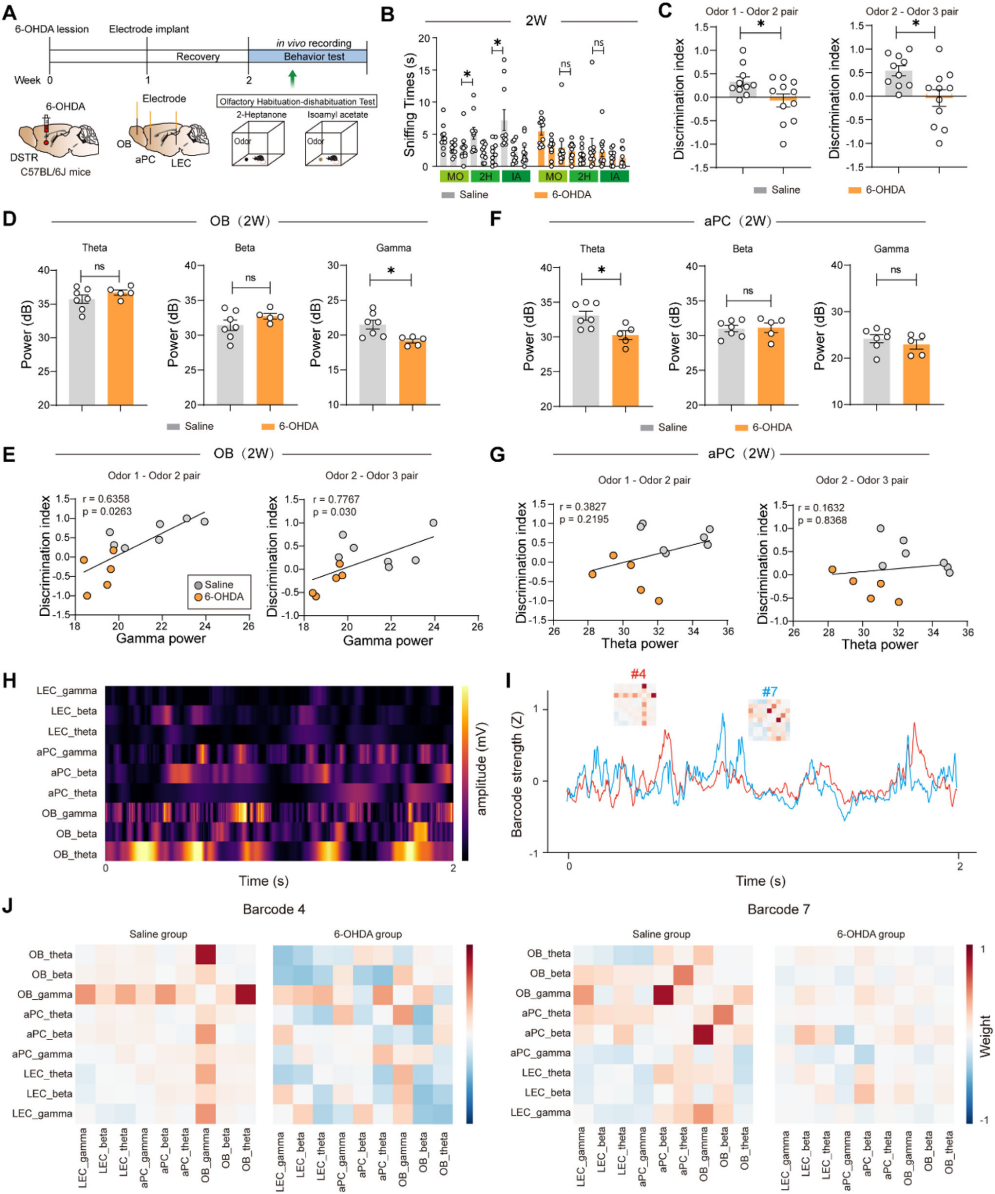
Gamma oscillations mediate odor discrimination deficits in early‐stage PD. A) Schematics and timeline of the experimental procedures. Behavioral and LFP recordings are conducted simultaneously two weeks after 6‐OHDA injection. B,C) Quantification of the time spent sniffing different odor‐infused cotton swabs during the olfactory habituation‐dishabituation test (B), and assess the preference for each novel odor (C). Saline: n = 10 mice, 6‐OHDA: n = 11 mice. D) Quantification of LFP power (3–95 Hz) in the OB recorded during the olfactory habituation–dishabituation test. Saline: n = 7 mice, 6‐OHDA: n = 5 mice. E) Pearson's correlation analysis of the discrimination index versus OB‐gamma power. Saline: n = 7 mice, 6‐OHDA: n = 5 mice. F) Quantification of LFP power (3‐95 Hz) in the aPC. Saline: n = 7 mice, 6‐OHDA: n = 5 mice. G) Pearson's correlation analysis of the discrimination index versus aPC‐theta power. Saline: n = 7 mice, 6‐OHDA: n = 5 mice. H) Instantaneous amplitude time courses for each of the selected IMFs from each region. These amplitudes were normalized by their standard deviation to produce the amplitude time‐series matrix. I) Barcode odor discrimination scores. A higher score indicates that the barcode strength reflects the ability of odor discrimination. J) Barcode visualization of control and 6‐OHDA mice. Note that gamma‐band signal interactions dominate these barcodes. Data are presented as mean ± SEM; ^*^ *P* < 0.05; ns, not significant. Unpaired two‐tailed Student's t test for C,D,F) Pearson correlation analysis for E,G) One‐way ANOVA followed by Tukey's multiple comparisons for B.

To characterize olfactory dysfunction in the 6‐OHDA mouse model of PD, we employed an odor habituation–dishabituation paradigm (**Figure** **2**A). Control mice exhibited normal odor discrimination behavior, showing significant dishabituation when presented with novel Odor 2 following habituation to Odor 1 (Odor 1‐Odor 2 pair: discrimination index = 0.33 ± 0.10; Odor 2‐Odor 3 pair: discrimination index = 0.54 ± 0.11). In contrast, 6‐OHDA mice demonstrated markedly impaired olfactory discrimination (Odor 1‐Odor 2 pair: discrimination index = −0.05 ± 0.16; Odor 2‐Odor 3 pair: discrimination index = −0.02 ± 0.18 Figure 2B,C), indicating specific deficits in odor recognition memory. While NOR performance was not altered at early‐stage PD (Figure S3, Supporting Information). This behavioral impairment emerged at 2 weeks post‐injection, preceding the onset of motor symptoms and coinciding with the observed dopaminergic neuron loss in the glomerular layer of OB.

To establish the neural basis of olfactory impairment in PD model mice, we performed simultaneous multi‐regional electrophysiological recordings in the OB, anterior piriform cortex (aPC), and LEC during odor discrimination tasks. Power spectral analysis demonstrated that 6‐OHDA mice exhibited a reduction in gamma‐band power in the OB compared to controls (saline 21.49 ± 0.64 dB vs 6‐OHDA 19.16 ± 0.29 dB; *p* = 0.0152; Figure 2D), with gamma power showing a strong positive correlation with odor discrimination performance (Odor 1‐Odor 2 pair: r = 0.6358, *p* = 0.0263; Odor 2‐Odor 3 pair: r = 0.7767, *p* = 0.03; Figure 2E). In contrast, while theta‐band power in the aPC was reduced in 6‐OHDA mice (saline 33.07 ± 0.66 dB vs 6‐OHDA 30.26 ± 0.65 dB; *p* = 0.0149; Figure 2F), this reduction did not correlate with behavioral discrimination index (Odor 1‐Odor 2 pair: r = 0.3827, *p* = 0.2195; Odor 2‐Odor 3 pair: r = 0.1632, *p* = 0.8368; Figure 2G), indicating frequency‐specific and regionally distinct contributions to olfactory processing deficits in 6‐OHDA mice.

We next examined functional connectivity between olfactory regions by analyzing cross‐spectral coherence (CSC) between the OB‐aPC and OB‐LEC circuits. 6‐OHDA mice showed a reduction in gamma‐band OB‐aPC coherence compared to controls (saline 0.62 ± 0.026 vs 6‐OHDA 0.396 ± 0.017; *p* < 0.001; Figure S4A,B, Supporting Information), which strongly correlated with odor discrimination index (Odor 1‐Odor 2 pair: r = 0.6942, *p* = 0.0122; Odor 2‐Odor 3 pair: r = 0.5895, *p* = 0.0437; Figure S4C, Supporting Information), paralleling the observed decrease in OB‐aPC phase‐amplitude coupling (saline 0.0034 ± 0.0003 vs 6‐OHDA 0.0017 ± 0.0002; *p* = 0.0018; Figure S4G–I, Supporting Information). In contrast, theta‐band OB‐LEC coherence increased in 6‐OHDA mice (saline 0.27 ± 0.012 vs 6‐OHDA 0.43 ± 0.011; *p* < 0.001; Figure S4D,E, Supporting Information), yet showed no behavioral correlation (Odor 1‐Odor 2 pair: r = −0.565, *p* = 0.0556; Odor 2‐Odor 3 pair: r = −0.3293, *p* = 0.2959; Figure S4F, Supporting Information). This frequency‐dependent dissociation demonstrates that gamma‐mediated OB‐aPC connectivity specifically supports olfactory cognitive function, while enhanced theta‐band OB‐LEC coupling may represent compensatory or pathological changes unrelated to odor discrimination performance.

Building upon behavior‐synchronized multiregional LFP recordings, we first extracted oscillatory signals across brain regions and performed pattern recognition of multiregional neural activity using an unsupervised analytical approach, designating these characteristic patterns as “Barcodes” (Figure 2H,I). Temporal and spatial correspondence analyses between distinct Barcodes and behavioral epochs revealed that Barcodes #4 and #7 in control mice demonstrated prominent gamma‐band activation during olfactory cognition. In contrast, 6‐OHDA mice exhibited significantly attenuated gamma oscillatory strength within corresponding Barcodes (Figure 2J). These results establish that gamma‐band‐organized neural Barcodes in olfactory networks represent a characteristic signature of functional olfactory processing. The selective impairment of gamma‐driven Barcode features in PD models supports the hypothesis that coordinated gamma oscillations serve as critical temporal codes for maintaining olfactory network integrity, while their degradation may mechanistically underlie early sensory deficits in Parkinsonian pathophysiology.

### 2.3 Theta Oscillations Dominate Novel Object Recognition in Late‐Stage PD

**Figure 3:**
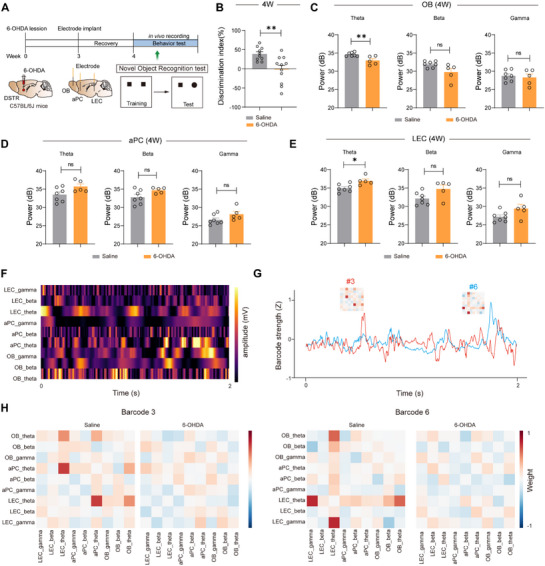
Theta oscillations dominate novel object recognition in late‐stage PD. A) NOR and LFP recordings are conducted simultaneously 4 weeks after 6‐OHDA injection. B) Analysis of new object preference for A. Saline: n = 10 mice, 6‐OHDA: n = 11 mice. C–E) Quantification of LFP power (3‐95 Hz) in the OB (C), aPC (D) and LEC (E) during NOR. Saline: n = 7 mice, 6‐OHDA: n = 5 mice. F) Instantaneous amplitude time courses for each of the selected IMFs from each region. These amplitudes were normalized by their standard deviation to produce the amplitude time‐series matrix. G) Barcode cognition scores. A higher score indicates that the barcode strength reflects the ability of cognitive function. H) Barcode visualization of control and 6‐OHDA mice. Note that theta‐band signal interactions dominate these barcodes. Data are presented as mean ± SEM; ^*^ *P* < 0.05, ^**^ *P* < 0.01; ns, not significant. Unpaired two‐tailed Student's t test for B‐E.

To investigate olfactory network alterations during late‐stage PD progression, neural oscillatory activity was recorded during NOR tasks (**Figure** **3**A). Longitudinal analysis of late‐stage PD progression revealed that 6‐OHDA mice exhibited significantly impaired novel object recognition (discrimination index: saline 44.53 ± 6.09% vs 6‐OHDA −2.50 ± 4.35%, *p* < 0.01; Figure 3B) at 4 weeks post‐injection, accompanied by distinct theta oscillatory disturbances characterized by a reduction in the olfactory bulb (saline 34.84 ± 0.54 dB vs 6‐OHDA 32.02 ± 0.45 dB; *p* < 0.01; Figure 3C) and an increase in the LEC (saline 34.49 ± 0.64 dB vs 6‐OHDA 37.16 ± 0.29 dB; *p* < 0.05; Figure 3E), while piriform cortex activity remained unchanged (Figure 3D), demonstrating that impaired NOR performance specifically correlates with dysregulated theta synchrony across the OB‐LEC network rather than localized cortical alterations. We further recorded neural oscillatory activity during olfactory habituation–dishabituation test at late‐stage PD, 6‐OHDA mice exhibited a significant drop of cross‐correlation at both gamma in OB‐aPC (Figure S5A–F, Supporting Information) and beta bands in OB‐LEC circuit (Figure S5G–L, Supporting Information). These results show that the 6‐OHDA mice have impairments in the amount and direction of information influx underlying sensory deficits.

Temporal and spatial correspondence analyses between distinct Barcodes and behavioral epochs revealed that Barcodes #3 and #6 in control mice demonstrated prominent theta‐band activation during NOR tasks (Figure 3F,G). In contrast, 6‐OHDA mice exhibited significantly attenuated theta oscillatory strength within corresponding Barcodes (Figure 3H). These findings indicate that theta‐band‐organized neural Barcodes in olfactory networks represent a characteristic signature of functional cognitive processing. The selective degradation of theta‐driven Barcode features in PD models suggests that spatiotemporally coordinated theta oscillations across brain regions may serve as a neural substrate for dynamic cognitive network integration.

### 2.4 Early‐Stage Cross‐Network Gamma Oscillations Predict Cognitive Impairment

**Figure 4:**
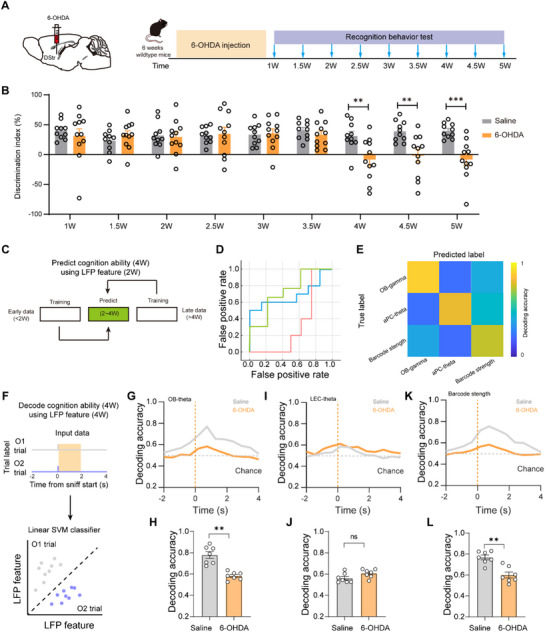
Early‐stage Cross‐network gamma oscillations predict cognitive impairment. A) NOR and LFP recordings are conducted simultaneously from 1 to 5 weeks after 6‐OHDA injection. B) Analysis of dynamics of new object preference. C) Sample workflow of the modeling approach. Data of the early LFP recordings (2 W) and the later NOR test (4W) is from the same cohort of animals. Saline: n = 10 mice, 6‐OHDA: n = 11 mice. D) AUC values achieved by the machine learning models on the testing dataset. E) The confusion matrices for the training and testing sets. F) Schematic of population decoding of familiar and novel object choices. A linear support vector machine (SVM) classifier was trained to distinguish between familiar versus novel object trials. G–L) Decoding accuracy of familiar and novel object choices by OB theta (G), LEC theta (I), and OB‐LEC barcode strength (K). Averaged decoding accuracy of familiar and novel object choices by OB theta (H), LEC theta (J), and OB‐LEC barcode strength (L). Saline: n = 7 mice, 6‐OHDA: n = 7 mice. Data are presented as mean ± SEM; ^**^ *P* < 0.01, ^***^ *P* < 0.001; ns, not significant. Unpaired two‐tailed Student's t test for B,H,J,L).

To investigate the dynamic of cross‐network interactions during PD progression, we performed simultaneous behavioral assessments and LFP recordings (**Figure** **4**A). Our longitudinal investigation of cross‐network dynamics in PD progression revealed that early‐stage (≤2 weeks) gamma‐band synchrony between olfactory and piriform cortex networks robustly predicted subsequent cognitive impairment at 4 weeks post‐injection (Figure 4B). Machine learning analysis based on a random forest model demonstrated that gamma oscillation recorded during the pre‐symptomatic phase could forecast cognitive decline with 89.6% accuracy, significantly outperforming traditional behavioral measures (Figure 4C–E). Notably, the predictive power of gamma synchrony peaked prior to observable cognitive deficits in the novel object recognition test, establishing cross‐network gamma oscillations as both a sensitive early biomarker and a mechanistic substrate of network‐level dysfunction in PD pathogenesis.

To determine how oscillatory features contribute to recognition choice decoding, we performed population‐level machine learning analyses by using a linear support vector machine (SVM) classifier to discriminate familiar object choice and novel object choice trials, the results revealed that linear classifiers trained on behavior‐aligned neural activity could predict novelty choices in control mice with 78.9% accuracy from OB theta oscillations and 76.1% accuracy from OB‐LEC Barcode strength, while these predictive capacities were completely abolished in 6‐OHDA mice (OB theta: 59.1%, *p* = 0.0064; OB‐LEC Barcode: 58.2%, *p* = 0.0074) despite intact LEC theta activity (control: 57.1% vs 6‐OHDA: 60.9%, *p* = 0.623; Figure 4F–L), demonstrating that PD‐related cognitive deficits specifically impair distributed theta‐rhythmic coordination across olfactory‐entorhinal networks rather than localized LEC activity, and highlighting the critical role of OB‐centered theta synchrony in novelty detection.

### 2.5 Chemogenetics Manipulations of OB‐LEC Circuit Reverse the Disrupted Cross‐Network Oscillation and Cognition Ability in PD Mice

**Figure 5:**
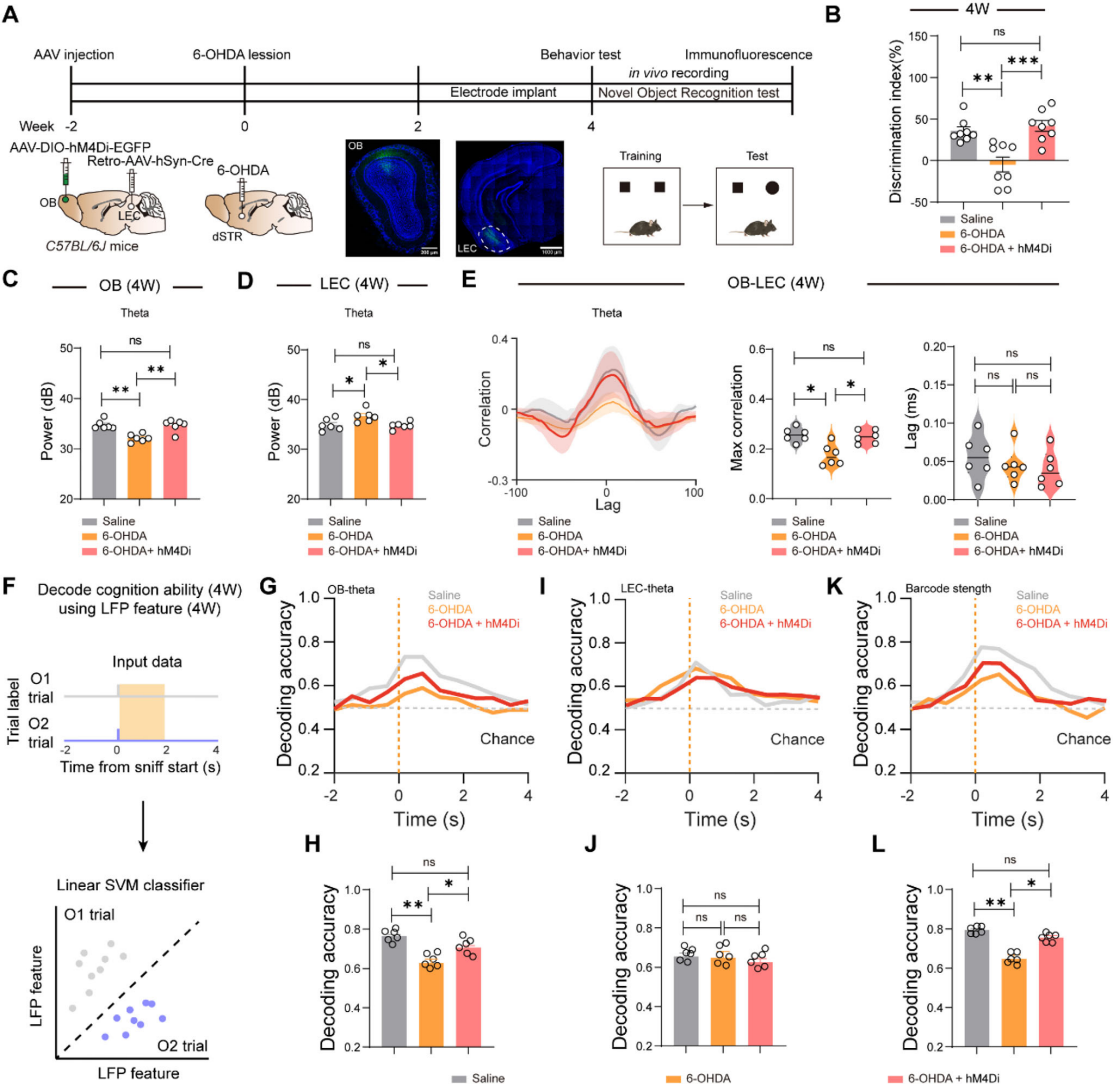
Chemogenetics manipulations of OB‐LEC circuit reverse the disrupted cross‐network oscillation and cognitive ability in PD mice. A) Schematic of chemogenetics manipulation and NOR and representative coronal sections showing virus expression in the OB (left) and retrograde labeling in LEC (right). B) Quantification of novelty preference in saline mice, 6‐OHDA mice, and 6‐OHDA + hM4Di mice. Saline: n = 8 mice, 6‐OHDA: n = 8 mice, 6‐OHDA + hM4Di: n = 8 mice. C,D) Quantification of theta power in OB, LEC in saline mice, 6‐OHDA mice, and 6‐OHDA + hM4Di mice. Saline: n = 6 mice, 6‐OHDA: n = 6 mice, 6‐OHDA + hM4Di: n = 6 mice. E) Correlation analysis between the OB and LEC in saline mice, 6‐OHDA mice, and 6‐OHDA + hM4Di mice. Saline: n = 6 mice, 6‐OHDA: n = 6 mice, 6‐OHDA + hM4Di: n = 6 mice. F) Schematic of population decoding of familiar and novel object choices. A linear support vector machine (SVM) classifier was trained to distinguish between familiar vs novel object trials. G–L) Decoding accuracy of familiar and novel object choices by OB theta (G), LEC theta (I), and OB‐LEC barcode strength (K). Averaged decoding accuracy of familiar and novel object choices by OB theta (H), LEC theta (J), and OB‐LEC barcode strength (L). Saline: n = 6 mice, 6‐OHDA: n = 6 mice, 6‐OHDA + hM4Di: n = 6 mice. Data are presented as mean ± SEM; ^*^ *P* < 0.05, ^**^ *P* < 0.01, ^***^ *P* < 0.001. ns, not significant. One‐way ANOVA followed by Tukey's multiple comparisons for B–D, E (right), H, J, L.

To better understand the effects of cross‐network activity in respect of cognition impairment, we inhibited M/T neurons by hM4Di (**Figure** **5**A). Our results indicated that chemogenetic inhibition of M/T neurons can rescue cognitive deficits in 6‐OHDA mice, restoring novel object discrimination performance (discrimination index: saline 41.3 ± 5.59% vs 6‐OHDA −8.4 ± 6.83% vs 6‐OHDA + hM4Di 52.7 ± 4.73%) and theta oscillations in both OB (saline 36.7 ± 0.73 dB vs 6‐OHDA 33.8 ± 0.53 dB vs 6‐OHDA + hM4Di 35.8 ± 0.61 dB) and entorhinal cortex (saline 34.9 ± 0.23 dB vs 6‐OHDA 36.9 ± 0.15 dB vs 6‐OHDA + hM4Di 35.2 ± 0.27 dB; Figure 5B–E), demonstrating that targeted modulation of OB‐LEC circuit activity can effectively reverse PD‐related cognitive impairment through restoration of cross‐network theta synchrony.

Population‐level decoding analysis demonstrated that chemogenetic inhibition of M/T neurons restored the predictive capacity of OB theta oscillations (saline 0.78 ± 0.07 vs 6‐OHDA 0.62 ± 0.11 vs 6‐OHDA + hM4Di 0.71 ± 0.06) and OB‐LEC Barcode strength (saline 0.79 ± 0.03 vs 6‐OHDA 0.64 ± 0.13 vs 6‐OHDA + hM4Di 0.75 ± 0.08) for novelty detection in 6‐OHDA mice (Figure 5F–L), while leaving LEC theta activity unchanged, confirming that targeted modulation of M/T neuron activity selectively rescues the distributed theta‐rhythmic coordination essential for cognitive function without affecting localized LEC processing.

## 3 Discussion

Our study establishes a frequency‐resolved temporal hierarchy of cross‐olfactory network dysfunction driving PD‐associated cognitive impairment, revealing that mitral cell hyperexcitability and gamma oscillation deficits across‐olfactory network precede and predict cognitive decline of late‐stage PD, while theta oscillation deficit across‐olfactory network encodes cognitive decline of late‐stage PD. This dual‐phase oscillatory disruption provides both predictive biomarkers (gamma) and severity indicators (theta), creating a physiologically grounded framework for staging PD pathology that bridges cellular excitability, network dynamics, and behavioral outcomes.

The observed mitral cell hyperexcitability and gamma oscillation reduction in the OB corroborate emerging evidence of early inhibitory circuit collapse in PD. M/T cells serve as the principal output neurons of the OB. They receive direct excitatory input from olfactory sensory neurons and convey processed neural information to higher olfactory centers.^[^ ^20^ ^]^ The observed deficits in odor discrimination may be explained by impaired neural signaling in the OB of PD mice, as evidenced by alterations in both spike activity and odor‐evoked gamma oscillations. Notably, our identification of phase‐amplitude dyssynchrony between OB and aPC activity mechanistically explains how olfactory sensory processing becomes decoupled,^[^ ^21^ ^]^ a phenomenon recently implicated in α‐synuclein‐mediated synaptic dysfunction.^[^ ^22^, ^23^ ^]^ This excitatory‐inhibitory imbalance may create a permissive environment for pathological protein propagation,^[^ ^24^ ^]^ as hyperactive mitral cells exhibit increased vulnerability to α‐synuclein oligomerization.^[^ ^25^ ^]^ While our study focused on circuit‐level alterations, recent single‐cell transcriptomic analyses reveal PD‐specific downregulation of parvalbumin interneuron markers in the OB, suggesting molecular substrates for gamma oscillation dysregulation that warrant further investigation.

The inverse correlation between LEC‐theta hyperactivity and cognitive performance challenges the conventional view of theta oscillations as uniformly supportive of memory processes.^[^ ^26^, ^27^, ^28^ ^]^ Our data support a model where excessive theta synchrony disrupts hippocampal‐entorhinal cross‐frequency coupling; a mechanism recently associated with spatial memory deficits in tauopathy models.^[^ ^29^ ^]^ Intriguingly, the stage‐specificity of theta abnormalities mirrors neuropathological staging studies showing delayed entorhinal tau deposition compared to early α‐synuclein pathology in olfactory regions.^[^ ^30^ ^]^ This temporal dissociation highlights the need to reevaluate oscillatory biomarkers within neuropathological context, as similar frequency bands may reflect distinct mechanisms depending on disease phase.

The complementary predictive (gamma) and prognostic (theta) values of our electrophysiological biomarkers align with growing consensus that multi‐scale, dynamic signatures outperform static pathological measures in neurodegenerative disease stratification.^[^ ^31^, ^32^, ^33^ ^]^ While our electrophysiological signatures show high predictive value, combining them with α‐synuclein CSF assays^[^ ^34^, ^35^, ^36^ ^]^ or DTI‐based glymphatic dysfunction metrics could enhance diagnostic precision.^[^ ^37^, ^38^, ^39^ ^]^ A critical next step will be to employ closed‐loop stimulation to restore gamma rhythms in sync with intrinsic OB‐aPC activity. This intervention would directly test whether gamma coherence mechanistically constrains subsequent cognitive decline. Our machine learning models identified non‐linear interactions between oscillatory biomarkers and motor symptom severity, supporting recent hypotheses about shared network vulnerabilities underlying PD's motor‐cognitive duality.^[^ ^40^ ^]^

Translating these oscillatory findings to human PD remains technically challenging, primarily due to the anatomical depth and small size of the human OB,^[^ ^41^ ^]^ which limits direct access to OB–LEC network activity. However, emerging non‐invasive approaches, such as nasal olfactory probes, intranasal or wearable EEG arrays, and high‐resolution MEG, are beginning to enable detailed tracking of olfactory network dynamics.^[^ ^42^ ^]^ Future clinical studies combining these modalities with CSF α‐synuclein assays or functional connectome analyses could help bridge this translational gap, establishing whether early deficits in OB gamma oscillations represent viable biomarkers of prodromal PD. Longitudinal monitoring using such tools may ultimately enable network‐targeted interventions to be deployed before irreversible neurodegeneration occurs.

By delineating the spatiotemporal hierarchy of oscillatory network failure in PD, this work bridges two critical gaps in neurodegeneration research: 1) the disconnect between cellular pathology and system‐level dysfunction, and 2) the clinical translation of basic rhythm physiology. The identification of phase‐specific network biomarkers not only advances PD stratification but also provides a template for investigating frequency‐domain pathophysiology in other proteinopathies. As the field moves toward circuit‐informed therapeutic strategies, our findings underscore the imperative to target oscillatory network properties before irreversible structural degeneration occurs, a paradigm shift supported by recent successes in cognitive rescue through rhythm restoration.^[^ ^43^, ^44^, ^45^ ^]^

## 4 Limitations of the Study

Several underexplored axes demand prioritization: First, whether the observed deficits in OB‐aPC gamma oscillations play a causal role in early olfactory dysfunction or merely represent an epiphenomenon remains an open question. To address this, future studies using optogenetic entrainment of OB gamma rhythms during the prodromal stage could help determine whether restoring physiological synchrony rescues olfactory discrimination and delays subsequent network deterioration. Second, the therapeutic time window for rhythm‐specific neuromodulation requires definition via longitudinal studies in prodromal cohorts, ideally integrating wearable EEG with fluid biomarkers. Third, the observed phenotypes in 6‐OHDA model may not solely result from dopaminergic neuron depletion, as it is possible that noradrenergic and serotonergic systems are also compromised.^[^ ^46^ ^]^ Employing desipramine‐pretreated models in future work will help distinguish the specific contribution of dopaminergic dysfunction to the olfactory‐cognitive impairments.

## 5 Conclusion

We propose that cross‐olfactory network oscillations serve as modifiable targets for addressing cognitive decline in Parkinson's disease. Our study reveals a dual‐phase dysfunction: early‐stage mitral cell hyperexcitability and gamma‐band deficits precede and predict late‐stage cognitive impairment, while theta‐band disturbances correlated with its severity. By integrating in vivo electrophysiology, circuit‐specific pharmacology modulation, and machine learning‐based decoding, we identify a spatiotemporal hierarchy in which early gamma anomalies propagate into delayed theta‐mediated deficits. These finding establish a causal framework for sensory‐cognitive network disintegration in PD, providing physiologically predictive biomarkers (gamma oscillations for early detection), severity indicators (theta oscillations for staging), and mechanistic insights to guide clinical neuromodulation strategies.

## 6 Experimental Section

### Animals

Animal procedures were approved by the Animal Advisory Committees at Nanjing Medical University for the Care and Use of Laboratory Animals and conformed to the National Institutes of Health Guide for the Care and Use of Animals in China (IACUC‐1912036). Male *C57BL/6J* mice aged 6‐weeks were obtained from Shanghai SLAC Laboratory Animal Co., Ltd. (Shanghai, China). Animals were housed under standardized conditions, including a 12‐hour light/dark cycle, an ambient temperature of 22 ± 1 °C, and relative humidity between 40% and 70%. Food and water were provided ad libitum.

### 6‐OHDA Model Mice

Following a one‐week acclimation period, the mice underwent 6‐OHDA lesion modeling as described previously.^[^ ^47^ ^]^ Mice were anesthetized with isoflurane and secured in a stereotaxic frame, followed by injection of 1 µL of 6‑OHDA (3 µg/µL) into the dorsal striatum (coordinates: AP +0.50 mm; ML +1.50 mm; DV −3.00 mm). The 6‑OHDA stock solution (Cat. O879851; Shanghai Macklin Biochemical Technology Co., Ltd.) was prepared in 0.9% NaCl containing 0.2 mg/mL L‑ascorbic acid to prevent oxidation. Behavioral experiments were conducted 14 days after the 6‑OHDA injection.

### Rotarod Test

The rotarod test was employed to evaluate motor coordination and balance in mice.^[^ ^48^ ^]^ On Day 1, mice underwent three training trials on a rotating rod at a constant speed of 4 r/min for 10 min per trial. On Day 2, training continued with the rod accelerating from 4 to 15 r/min over a 10‐minute period, with three trials per mouse. On Day 3, the testing phase was conducted, during which the rotation speed increased linearly from 4 to 40 r/min within the first 5 min and then continued at this speed for a total duration of 10 min. Each mouse completed three independent trials. All sessions were video‐recorded, and latency to fall was measured using a stopwatch.

### Novel Object Recognition Test

The novel object recognition test leverages rodents’ innate preference for novelty to assess learning and memory function. During the habituation phase, two identical objects were placed at positions one‐quarter of the chamber length from the wall. Mice were positioned at the center of the chamber and allowed to freely explore the objects for 10 min. Following a 2‐hour rest period in their home cages, the testing phase began, during which one of the familiar objects was replaced with a novel one. Mice were then reintroduced into the chamber for an additional 5‐minute exploration session. A video tracking system was used to monitor and quantify exploratory behavior, with particular emphasis on the time spent sniffing each object. Simultaneous in vivo electrophysiological recordings were performed throughout to capture neural activity.

For the novel object recognition task, discrimination index = (Exploration time for novel object – Exploration time for familiar object) / (Exploration time for novel object + Exploration time for familiar object)

### Buried Food‐Seeking Test

The buried food‐seeking test was utilized to evaluate olfactory function in mice.^[^ ^49^ ^]^ Clean cages (28 cm × 13 cm × 17 cm) were prepared with a 3 cm‐thick layer of bedding material covering the bottom. A single food pellet was randomly buried at a depth of 3 cm in one corner of each cage. Prior to testing, mice were food‐deprived for 24 h. The entire procedure was video‐recorded, and the latency to locate the buried food pellet was measured.

### Habituation–Dishabituation Test

During the habituation phase, a cotton swab soaked in mineral oil was inserted into a side port of the testing chamber for 2 minutes, followed by a 2‐minute inter‐trial interval. This sequence was repeated three times to establish baseline olfactory exposure. In the subsequent dishabituation phase, the odorant was switched to 2‐heptanone, and the same presentation protocol was repeated three times, followed by three additional trials using isoamyl acetate. The entire procedure was video‐recorded to quantify sniffing duration during each odor presentation. Simultaneously, in vivo electrophysiological recordings were conducted to monitor neural activity corresponding to each phase, allowing for an integrated assessment of both behavioral and circuit‐level olfactory responses.

For the habituation–dishabituation test, discrimination index = (Exploration time for novel odor – Exploration time for familiar odor) / (Exploration time for novel odor + Exploration time for familiar odor).

### Immunohistochemistry

Brain tissue processing and immunofluorescence staining were conducted as previously described.^[^ ^50^ ^]^ Briefly, brain sections were cut at a thickness of 40 µm. The sections were incubated with a primary antibody anti‐TH (1:500, Millipore, Cat# AB152) for 48 h at 4 °C. After primary incubation, the sections were incubated with secondary antibody conjugated to Alexa Fluor 488 (1:300). Then the sections were performed with 4’,6‐diamidino‐2‐phenylindole (DAPI; Sigma, D8417) for 10 min. Fluorescence images were acquired using a Zeiss LSM 800 confocal laser scanning microscope.

### Stereotaxic Injection

Stereotaxic injection procedures were conducted following previously established methods.^[^ ^51^ ^]^ For the chemogenetic manipulation, pAAV2/9‐EF1α‐DIO‐hM4Di‐EGFP‐WPRE (5.21 × 10^13^ particles/ml) or pAAV2/9‐EF1α‐DIO‐EGFP‐WPRE (1.21 × 10^13^ particles/ml, Obio Technology, Shanghai, China) was microinfused unilaterally into the ventral OB of 6‐week‐old *C57BL/6J* mice. For the retrograde tracing, AAV2/R‐hSyn‐Cre (5.32 × 10^12^ particles/ml) (Brain VTA Co., Ltd. Wuhan, China) was microinfused unilaterally into the LEC (AP: −3.58 mm, ML: −3.85 mm, DV: −4.50 mm) of 6‐weeks‐old *C57BL/6J* mice.

The viral vectors were microinfused bilaterally at 200 nl per side, delivered at a rate of 50 nl/min using glass pipettes through a stereotaxic device (RWD Life Science).

### Electrode Implantation

Electrodes were implanted into three brain regions: OB (AP: +4.28 mm, ML: −1.00 mm, DV: −1.75 mm), aPC (AP: +2.10 mm, ML: −2.00 mm, DV: −4.50 mm), and LEC (AP: −3.58 mm, ML: −3.85 mm, DV: −4.50 mm) (*43*). Mice were allowed to recover for one week before the recording sessions. During data acquisition, electrodes were connected via a headstage to the CereCube system (NeuroXess). Spiking activity was recorded at a sampling rate of 40 kHz with a band‐pass filter of 300–5000 Hz. LFPs were sampled at 1000 Hz.

### In Vivo Electrophysiological Data Analysis

Spectral power in the theta (3–12 Hz), beta (15–35 Hz), and gamma (36–95 Hz) frequency bands was extracted using the bandpower function in MATLAB.^[^ ^52^ ^]^ The power spectral density was calculated using pwelch function.

Cross spectral coherence was applied to assess the frequency‐domain correlations between LFP from different brain regions. To evaluate synchrony within OB–LEC, both correlation and maximum cross‐correlation analyses were conducted. Analysis of OB–LEC network synchronization using correlation and maximum cross‐correlation. The xcorr function with the “coeff” option was used to compute the normalized time‐lagged cross‐correlation between OB and LEC. The absolute maximum of the cross‐correlation curve was extracted as an index of coupling strength.

“Barcode” extracts oscillatory signals from the LFPs of OB, aPC, and LEC.^[^ ^53^ ^]^ Through masked empirical mode decomposition, these signals are decomposed into intrinsic mode functions. The instantaneous amplitudes of the IMFs are calculated and standardized. Then, the standardized amplitudes of the IMFs from different brain regions are combined into a vector. A c‐participation vector is constructed through the outer product of this vector. A feature matrix is formed by sampling the co‐participation vectors every 250 ms, and independent component analysis is applied to this matrix. The resulting weight vectors are the “Barcodes”. By taking the dot product of these weight vectors and the co‐participation time‐series matrix of the non – standardized amplitude matrix, the strength values of each “Barcode” at different time points are obtained, which are used to analyze the coordinated patterns of multi – regional neuronal activities.

Spike sorting was performed using Offline Sorter, which was classified via principal component analysis.^[^ ^54^ ^]^ LFPs were band‐pass filtered, and the Hilbert transform was applied to obtain instantaneous phases. Each spike was aligned to the phase of the corresponding frequency band to determine firing phases. Phase‐locking was assessed by analyzing the spike phase distribution; neurons with significantly non‐uniform distributions were considered phase‐locked.

### Machine Learning Analysis

A linear kernel support vector machine (SVM) was applied to classify whether neural activity patterns were associated with interaction with a familiar object vs a novel object. The classifier was trained using neural signatures extracted during the task phase, with social choice decoding performance metrics serving as input features. The dataset was divided such that a randomly selected 80% of mice from both the control and 6‐OHDA lesion groups formed the training set, with the remaining 20% reserved for testing. To address potential class imbalance, the training set was balanced by equalizing sample sizes across groups. The training and testing procedure was repeated over 500 independent cross‐validation iterations to ensure robustness.

To evaluate the statistical significance of the decoding performance, we compared the mean accuracy obtained from these 500 cross‐validation runs against a chance‐level interval. This chance interval was defined as ±2 standard deviations around the theoretical chance level of 0.5, calculated from a null distribution of accuracies generated by 100 iterations of label shuffling.

### Quantification and Statistical Analysis

Mice were excluded from the study if subsequent examination revealed misplacement of electrodes, or if AAV transgene expression was absent or improperly located. Data were analyzed using GraphPad Prism. Prior to statistical testing, data were assessed for normality and homogeneity of variance. Two‐tailed t‐tests were used for comparisons between two groups when data met normality assumptions; otherwise, the Wilcoxon rank‐sum test was applied. For comparisons involving three or more groups, one‐way ANOVA was employed, and two‐way ANOVA was used when multiple factors were considered. Results are expressed as mean ± standard error of the mean. In all figures: ^*^P < 0.05, ^**^P < 0.01, ^***^P < 0.001, ^****^P < 0.0001.

## Conflict of Interest

The authors declare no conflict of interest.

## Author Contributions

S.W., Z.C., and X.M. contributed equally to this work. Z.C., S.W., and X.M. performed all experiments, analyzed the data, and wrote the manuscript. Y.P. and H.S. assisted with in vivo electrophysiology. J.C. and X.W. assisted with behavior tests. Y.Z., X.L., M.L., G.H., and T.S. reviewed the paper. X.L., Y.L., and F.H. designed the study, interpreted results, and reviewed the paper.

## Supporting information



Supporting Information

Supporting Table

## Data Availability

The data that support the findings of this study are available from the corresponding author upon reasonable request.
