# Supplementary material for: Olfactory‐to‐Entorhinal Network Dysrhythmias Drive Parkinson's Cognitive Impairment Through Frequency‐Specific Oscillatory Decoupling
Source: Adv Sci (Weinh). 2025 Dec 5;13(9):e12183. doi: 10.1002/advs.202512183 (PMC12904074; doi:10.1002/advs.202512183)
Supplement: Supplementary file 1 — Supporting Information [file ADVS-13-e12183-s002.docx]

Supplementary Materials for

**Olfactory-to-Entorhinal Network Dysrhythmias Drive Parkinson's Cognitive Impairment Through Frequency-Specific Oscillatory Decoupling**

Shuaishuai Wang *et al.*

Corresponding author: [fenghan169@njmu.edu.cn,](mailto:fenghan169@njmu.edu.cn) [lufx@njmu.edu.cn](mailto:lufx@njmu.edu.cn)

# This PDF file includes:

Figs. S1 to S5

**Figure. S1**


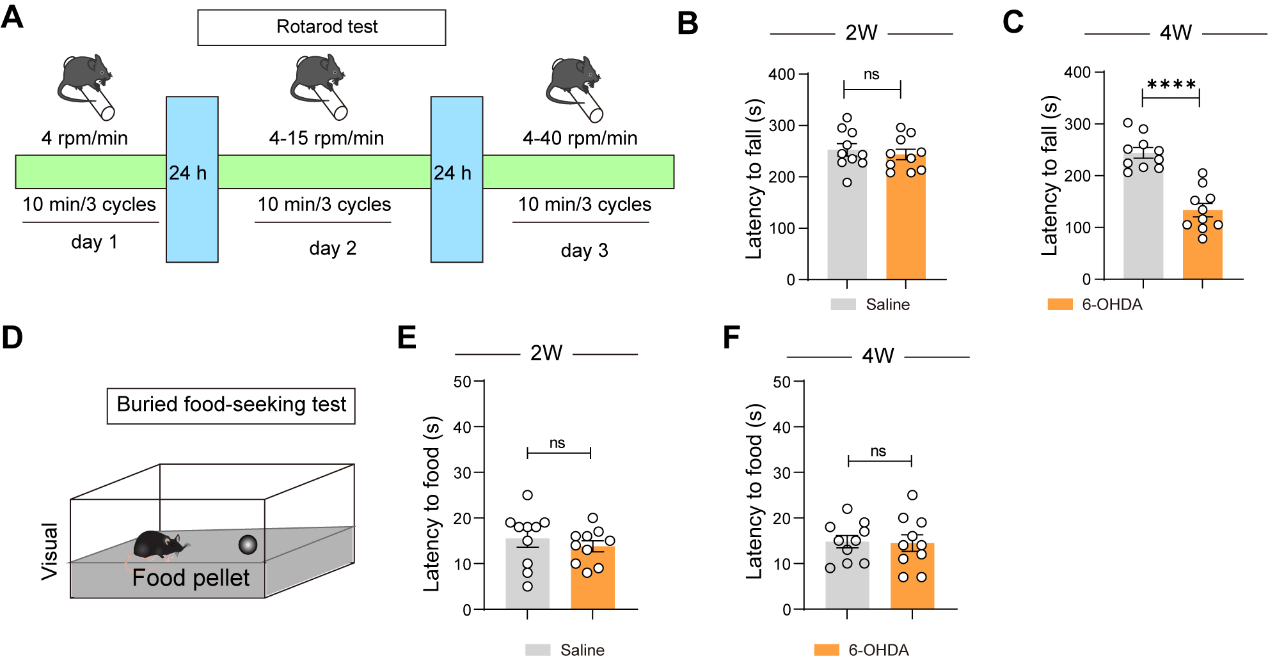


**Figure. S1 Moter impairment emerged at 4 weeks in PD mice.** **A**. Schematic illustrations of behavioral procedures for rotarod test. **B-C**. Analysis of latency to fall during rotarod test, Saline: n = 10 mice, 6-OHDA: n = 10 mice. **D**. Schematic illustrations of behavioral procedures for buried food-seeking test. **E-F**. Analysis of latency to find food 2 (E) and 4 (F) weeks after 6-OHDA injection. Saline: n = 10 mice, 6-OHDA: n = 10 mice. Data are presented as mean ± SEM; *^**^P* < 0.01; ns, not significant. Unpaired two-tailed Student’s t test for B,C, E,F.

**Figure. S2**


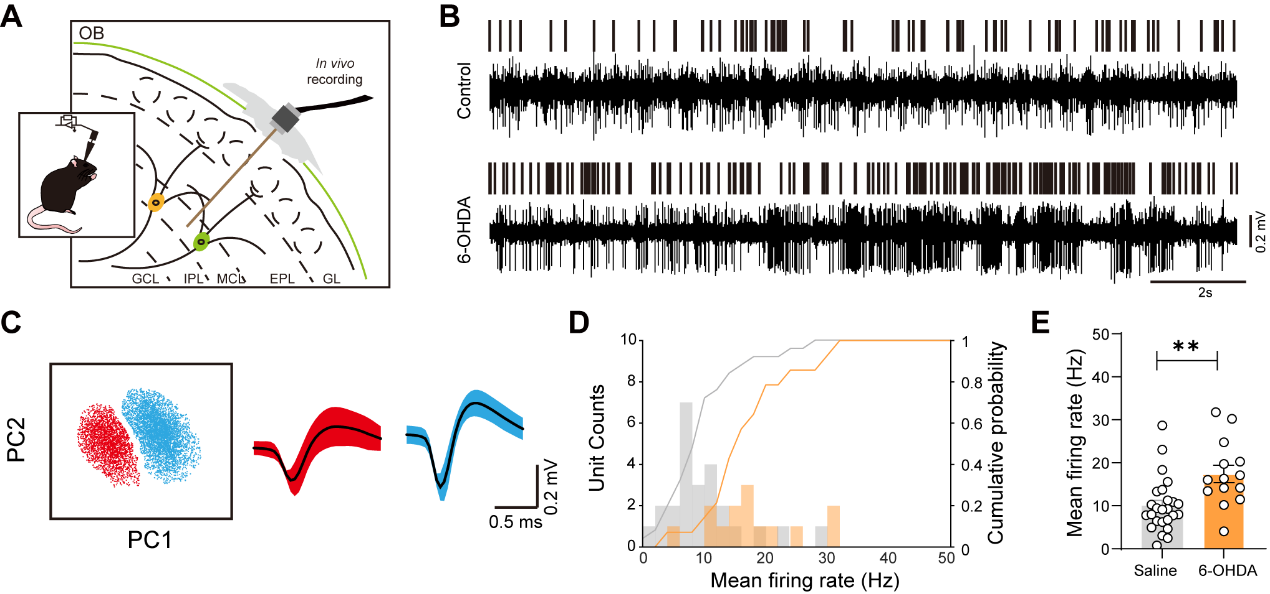


**Figure. S2 PD mice showed increased activity in OB.** **A**. Schematic illustrations of in vivo large-scale recording. **B**. Detected spikes and raw electrophysiological data. **C**. Schematic illustrations of spike waveform clustering. **D-E**. Analysis of firing rate of mitral cells in OB. Saline: n = 26 units from 5 mice, 6-OHDA: n = 15 units from 4 mice. Data are presented as mean ± SEM; *^**^P* < 0.01; ns, not significant. Unpaired two-tailed Student’s t test for E;

**Figure. S3**


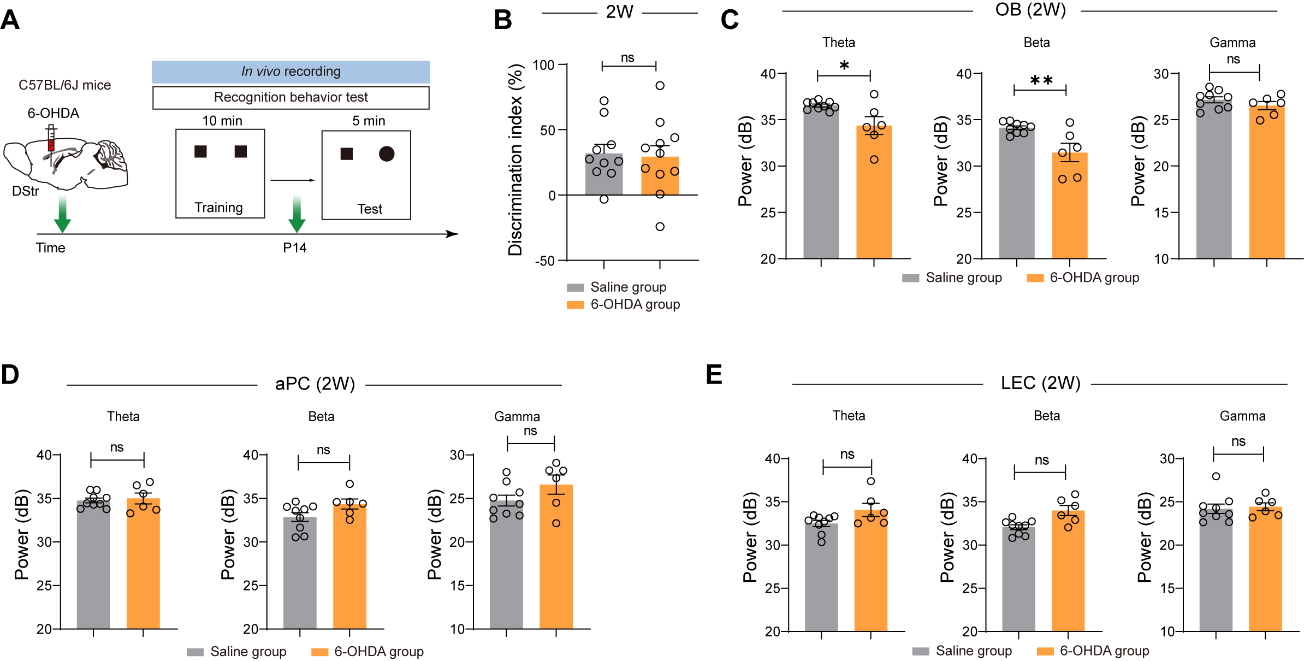


**Figure. S3 Novel object recognition deficits did not emerge at early-stage PD. A**. NOR and LFP recordings are conducted simultaneously 4 weeks after 6-OHDA injection. **B**. Analysis of new object preference for NOR test. Saline: n = 10 mice, 6-OHDA: n = 11 mice. **C-E**. Quantification of LFP power (3-95 Hz) in the OB (C), aPC (D) and LEC (E) during NOR test. Saline: n = 7 mice, 6-OHDA: n = 5 mice. Data are presented as mean ± SEM; *^*^P* < 0.05. ns, not significant. Unpaired two-tailed Student’s t test for B-E.

**Figure. S4**


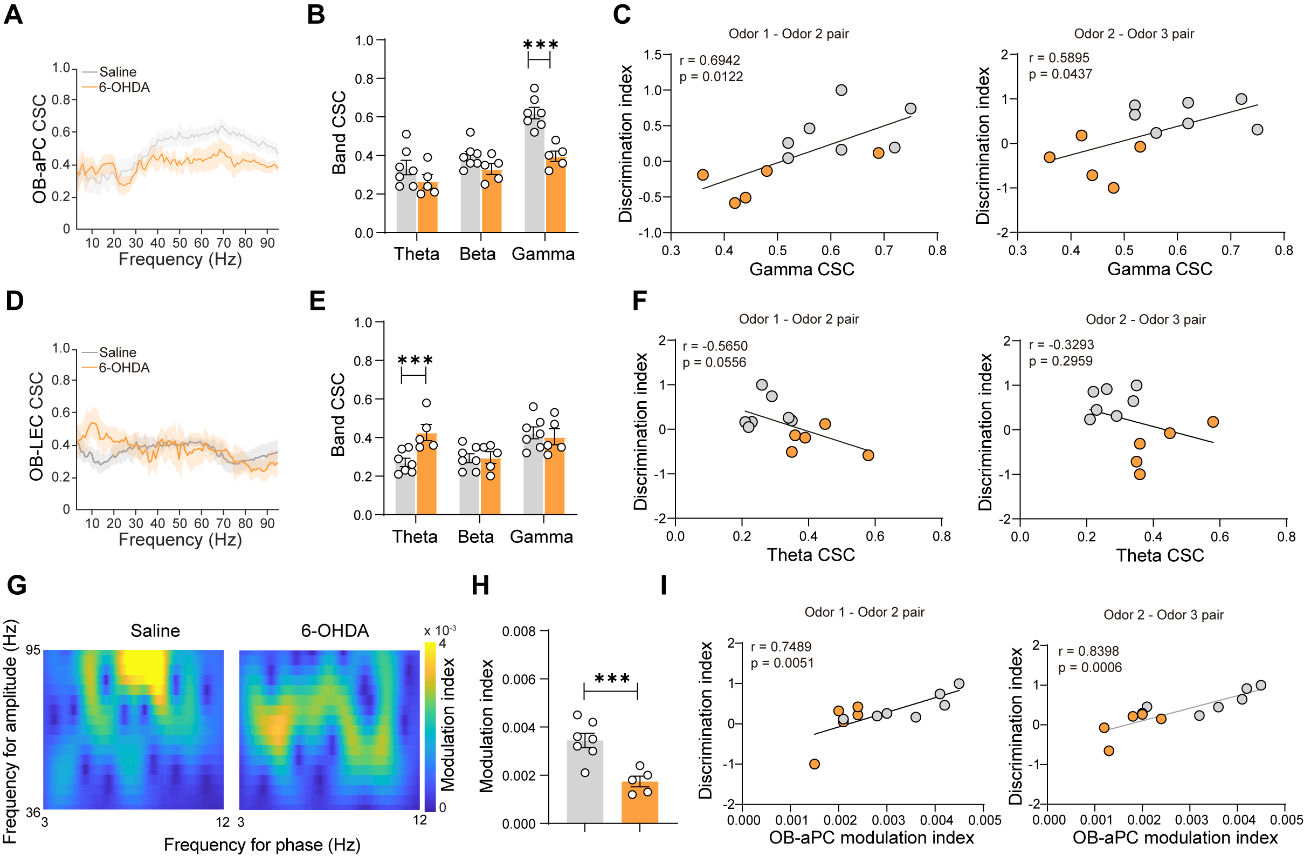


**Figure. S4 Olfactory dysfunction is associated with altered coherence in the olfactory regions at 2 weeks.** **A-B**. Analysis of OB-aPC cross-spectral coherence. Saline: n = 7 mice, 6-OHDA: n = 5 mice. **C**. Pearson’s correlation analysis of discrimination index versus OB-aPC gamma CSC. Saline: n = 7 mice, 6-OHDA: n = 5 mice. **D-E**. Analysis of OB-LEC cross-spectral coherence. Saline: n = 7 mice, 6-OHDA: n = 5 mice. **F**. Pearson’s correlation analysis of discrimination index versus OB-LEC theta CSC. Saline: n = 7 mice, 6-OHDA: n = 5 mice. **G-H**. Standard PAC comodulogram depicting the extent of amplitudes of oscillations at frequencies ranging from 36 to 95 Hz being modulated by the phase of oscillations at frequencies ranging from 3 to 12 Hz in during the sniffing period (G). Average PAC-modulation index in different relatively high-frequency bands coupled to fixed theta bands (H). Saline: n = 7 mice, 6-OHDA: n = 5 mice. **I**. Pearson’s correlation analysis of discrimination index versus OB-aPC modulation index. Saline: n = 7 mice, 6-OHDA: n = 5 mice. Data are presented as mean ± SEM; *^***^P* < 0.001; ns, not significant. Two-way repeated-measures ANOVA with Tukey’s post-hoc test for B,E; Unpaired two-tailed Student’s t test for H; Pearson correlation analysis for C,F,I.

**Figure. S5**


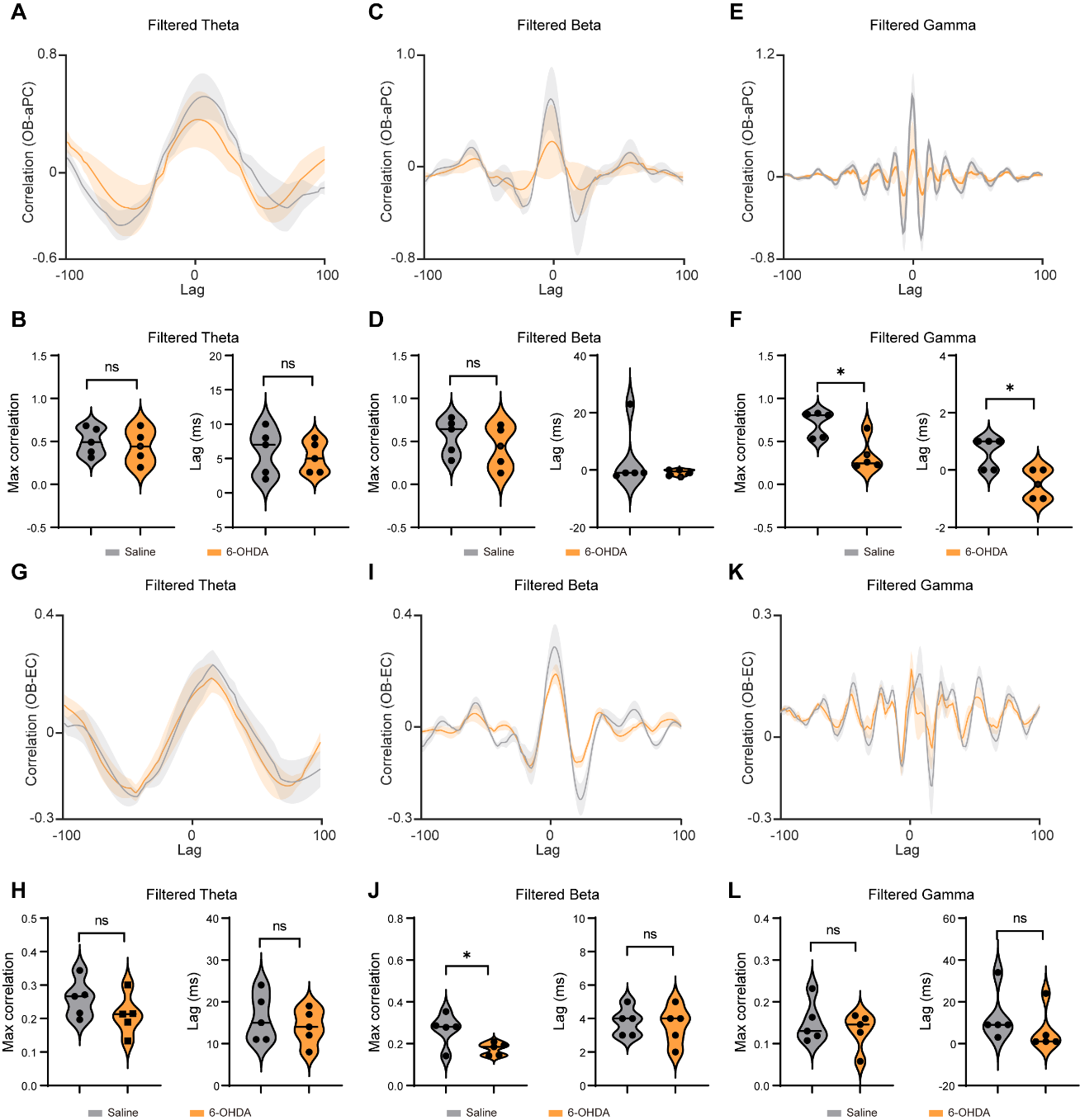


**Figure. S5. Disrupted synchrony in the OB-aPC-LEC circuit during olfactory habituation-dishabituation test at late-stage PD**. **A-F.** Mean correlation of OB-aPC in time lag at theta (A), beta (C), and gamma bands (E), respectively. Analysis of max correlation of OB-aPC at theta (B), beta (D), and gamma bands (F), respectively. Saline: n = 5 mice, 6-OHDA: n = 5 mice. **G-L.** Mean correlation of OB-LEC in time lag at theta (G), beta (I), and gamma bands (K), respectively. Analysis of max correlation of OB-LEC at theta (H), beta (J), and gamma bands (L), respectively. Saline: n = 5 mice, 6-OHDA: n = 5 mice. Data are presented as mean ± SEM; *^*^P* < 0.05; ns, not significant. Unpaired two-tailed Student’s t test for B,D,F,H,J,L.
